# Pulmonary Nodules in Patients With Nonpulmonary Cancer: Not Always Metastases

**DOI:** 10.1200/JGO.2015.002089

**Published:** 2016-02-03

**Authors:** Rafael Caparica, Milena Perez Mak, Claudio Henrique Rocha, Pedro Henrique Isaacsson Velho, Publio Viana, Mauricio R.L. Moura, Marcos Roberto Menezes, Marcelo B.P. Amato, Olavo Feher

**Affiliations:** **Rafael Caparica, Milena Perez Mak, Claudio Henrique Rocha, Pedro Henrique Isaacsson Velho, Publio Viana, Mauricio R.L. Moura, Marcos Roberto Menezes, and Olavo Feher**, Universidade de São Paulo; and **Marcelo B.P. Amato**, Faculdade de Medicina da Universidade de São Paulo, São Paulo, Brazil.

## Abstract

**Introduction:**

The differential diagnosis of pulmonary nodules (PNs) includes metastases, lung cancers, infectious diseases, and scar tissue, among others. Because data regarding whether and when to perform a PN biopsy in patients with cancer are scarce, clinicians tend to assume that PNs are metastatic disease based solely on imaging. The current study evaluated the findings of PN biopsies in a population of patients with cancer and sought to determine the variables that correlated with higher odds of metastatic disease.

**Patients and Methods:**

We conducted a retrospective, single-institution study that included consecutive patients with nonpulmonary solid malignancies who underwent PN biopsy from January 2011 to December 2013. Imaging and clinical variables were analyzed by logistic regression to determine the correlation between such variables and the odds of metastatic disease. Patients with previously known metastatic disease or primary hematologic malignancies were excluded.

**Results:**

Two hundred twenty-eight patients were included in the study. Metastatic disease was found in 146 patients (64%), 60 patients (26.3%) were diagnosed with a second primary lung tumor, and 22 patients (9.6%) had no cancer on biopsy. On multivariate analysis, the presence of multiple PNs (> 5 mm) and cavitation/necrosis were the only variables associated with higher odds (*P* < .05) of metastatic disease. We registered six (2.6%) procedure complications demanding active interventions, and no procedure-related death occurred.

**Conclusion:**

Multiple PNs (> 5 mm) and cavitation were the two characteristics associated with the highest chances of metastatic disease. Our findings demonstrate that PNs should not be assumed to be metastases without performing a biopsy. This assumption may lead to high rates of misdiagnosis. Tissue sampling is fundamental for accurately diagnosing patients with cancer.

## INTRODUCTION

Pulmonary nodules (PNs) are frequently encountered on imaging studies and represent a diagnostic challenge.^1^ Among patients already diagnosed with cancer, data regarding optimal investigation of PNs are scarce.^2-4^ Usually, the emergence of PNs during treatment or follow-up leads clinicians to favor the hypothesis that disease has metastasized to the lungs. In some malignancies, such as colorectal cancer and osteosarcoma, patients with few pulmonary metastases and good control of the primary site are considered for surgical resection.^2^ However, most patients thought to have pulmonary metastases are deemed incurable and are assigned to palliative treatment. This assumption directly affects the treatment and prognosis of patients.

There is no consensus or guideline regarding investigation of a PN in patients with extrapulmonary malignancies, and clinicians are commonly challenged to balance the benefit of obtaining tissue sampling for accurate diagnosis with the potential risks involved in a pulmonary biopsy procedure. Retrospective data in populations of patients with cancer show a high frequency of benign lesions (up to 58%) and primary lung cancers (up to 50%) found after biopsies or surgery for PNs.^5-7^ Some characteristics, such as primary site, nodule size, the presence of concomitant extrapulmonary lesions on imaging studies, and multiple PNs, have been predictive of malignancy in previous studies.^5-7^ To estimate the prevalence of metastatic disease in patients with cancer presenting with a PN and evaluate the variables associated with higher odds of finding metastatic disease on biopsy, we conducted a retrospective study.

## PATIENTS AND METHODS

This retrospective, observational, single-institution study was approved by the institutional review board and local ethics committee of the Instituto do Câncer do Estado de São Paulo in Brazil. We reviewed electronic charts of all patients older than 18 years of age undergoing a PN percutaneous computed tomography (CT)-guided core biopsy at the Instituto do Cancer do Estado de São Paulo consecutively from January 2011 to December 2013. All biopsy specimens were referred to the pathology department and were reviewed by a pathologist. The results were registered uniformly and electronically in patients’ charts. All patients had a previous biopsy-proven diagnosis of a solid tumor and were not known to have metastatic disease.

Patients with the following characteristics were excluded from enrollment: no previously confirmed cancer diagnosis; primary lung cancer (because the objective of the majority of these biopsies was to obtain tissue sampling for mutational testing) or hematologic malignancy (less likely to present with PNs and could present a bias to our final analysis); and known metastatic disease, defined by biopsy of the metastatic site. Patients presenting with PNs and concomitant extrathoracic nodules in CT scans (eg, liver, bones) were included in the analysis, provided that they did not have biopsy-proven metastatic disease. Patients who received previous thoracic radiotherapy as a part of breast cancer adjuvant treatment were included in this analysis. We established 0.5 cm as the minimum diameter for a lesion to be considered as a PN. Postprocedure complications were evaluated and described.

At our institution, the standard practice is to perform PN biopsies on lesions ≥ 0.5 cm in patients without a diagnosis of metastatic disease. However, the final decision is made by the patient’s respective clinician. Some patients presenting with multiple new PNs or imaging study features suggestive of metastatic disease may not have undergone biopsy because of their clinician’s decision.

Patients were classified according to primary site: colorectal, head and neck, urologic (kidney, testicular, prostate, and bladder), gastrointestinal noncolorectal (esophageal, gastric, pancreatic, small bowel, and biliary tract), breast, melanoma, gynecologic (ovarian, endometrial, and cervical), others (sarcomas, thyroid cancer, squamous cell), and unknown primary.

In those cases in which the PN was found to be a metastasis from a primary breast cancer, a comparison between the immunohistochemical profiles of the primary tumor and the metastases was performed regarding estrogen receptors and progesterone receptors (PRs), using the Allred score.^8^ Human epidermal growth factor receptor 2 status was assessed by means of immunohistochemical analyses (with 3+ indicating positive status) or fluorescence in situ hybridization (with an amplification ratio ≥ 2.0 indicating positive status), or both.

### Statistical Analysis

Statistical analyses were performed using SPSS, version 21.0 (SPSS, Chicago, IL). We analyzed clinical and radiologic variables possibly associated with higher odds of finding metastatic disease on biopsy, including primary tumor site, location of the nodules (superior or inferior lobes), previous thoracic radiotherapy, time from cancer diagnosis until biopsy, concomitant extrathoracic nodules, smoking history, presence of cavitation or necrosis, single or multiple nodules, and size of the biopsied nodule. To estimate the relationship between those variables and the biopsy result (metastatic *v* nonmetastatic disease), a multivariable logistic regression analysis was performed. These results are presented as OR (odds ratio) with its respective 95% confidence interval. Significance was set at an alpha error of .05.

## RESULTS

### Patient Characteristics

**Fig 1:**
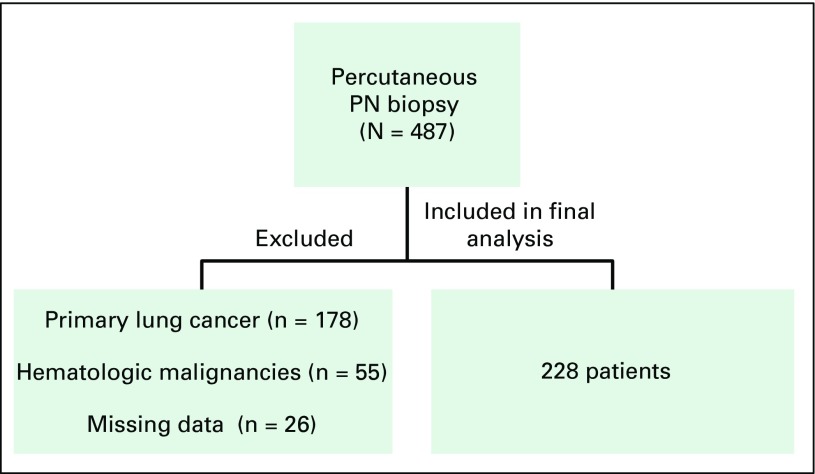
The data collection flowchart. PN, pulmonary nodule.

From January 2011 to December 2013, 487 patients underwent PN biopsies. After exclusion of 259 patients who did not meet the inclusion criteria, 228 were included in our final analysis. Patient characteristics are listed in Table 1, and the data collection flowchart is shown in Figure 1. Most patients were male (53.5%), colorectal cancer was the most common primary tumor site (25.8%), concomitant extrathoracic nodules were present in 25.9% of patients, multiple PNs were present in 50.9% of patients, and 30.3% of patients were never smokers.

**Table 1:**
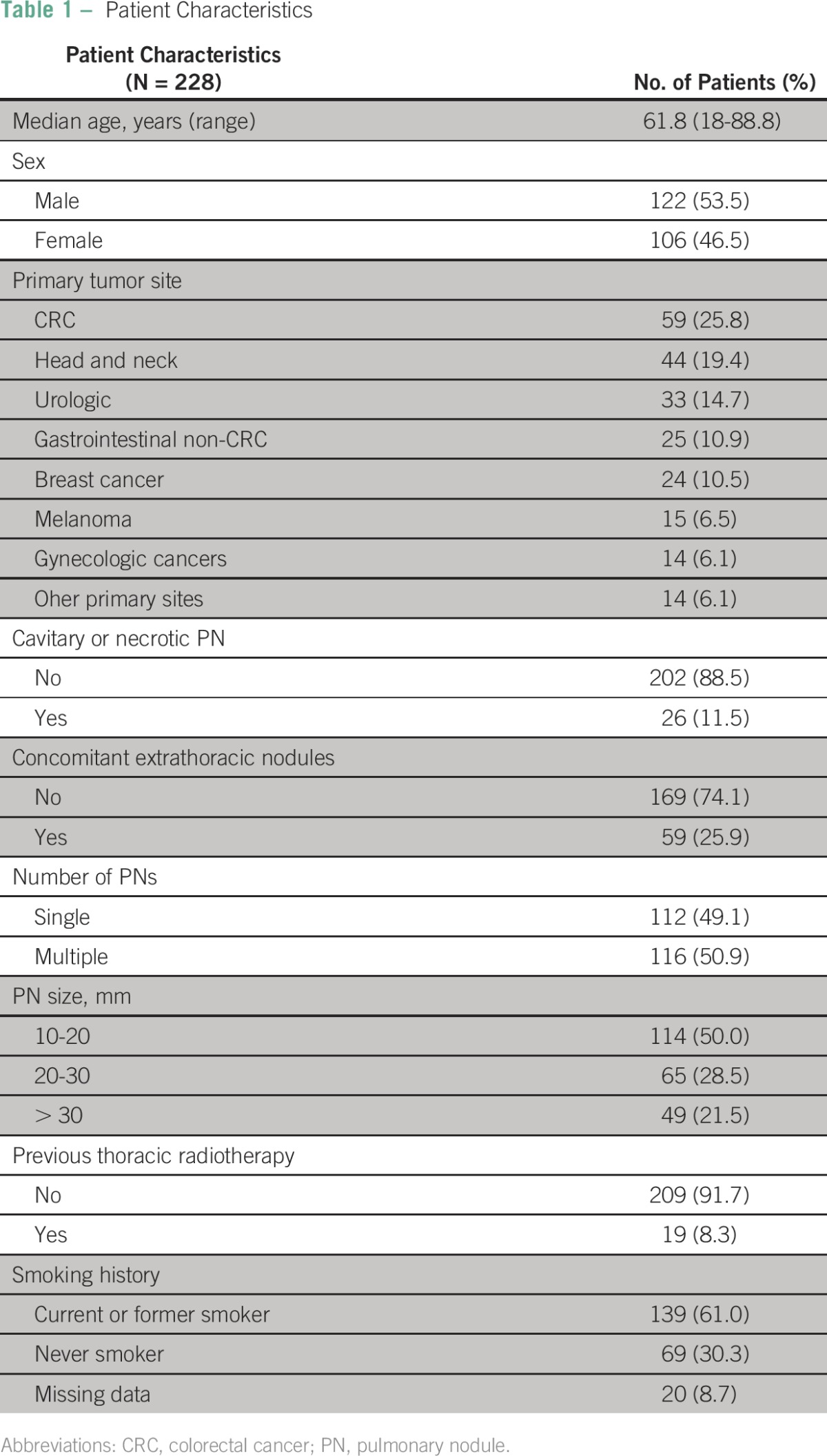
Patient Characteristics

### Biopsy Results

**Fig 2:**
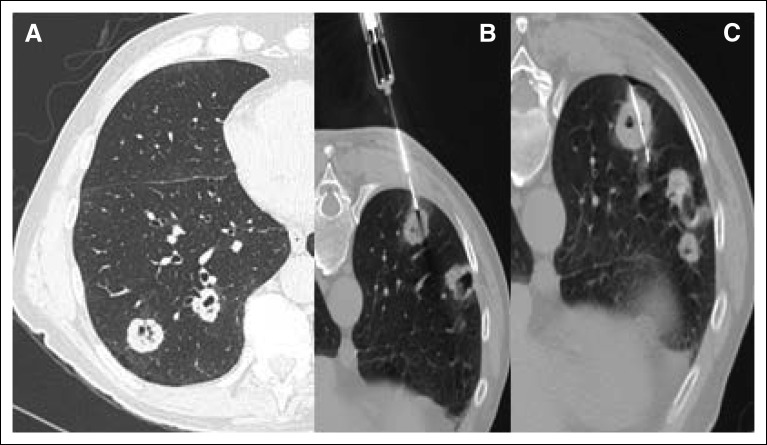
Computed tomography–guided pulmonary nodule biopsy performed on a patient included in the present study.

**Fig 3:**
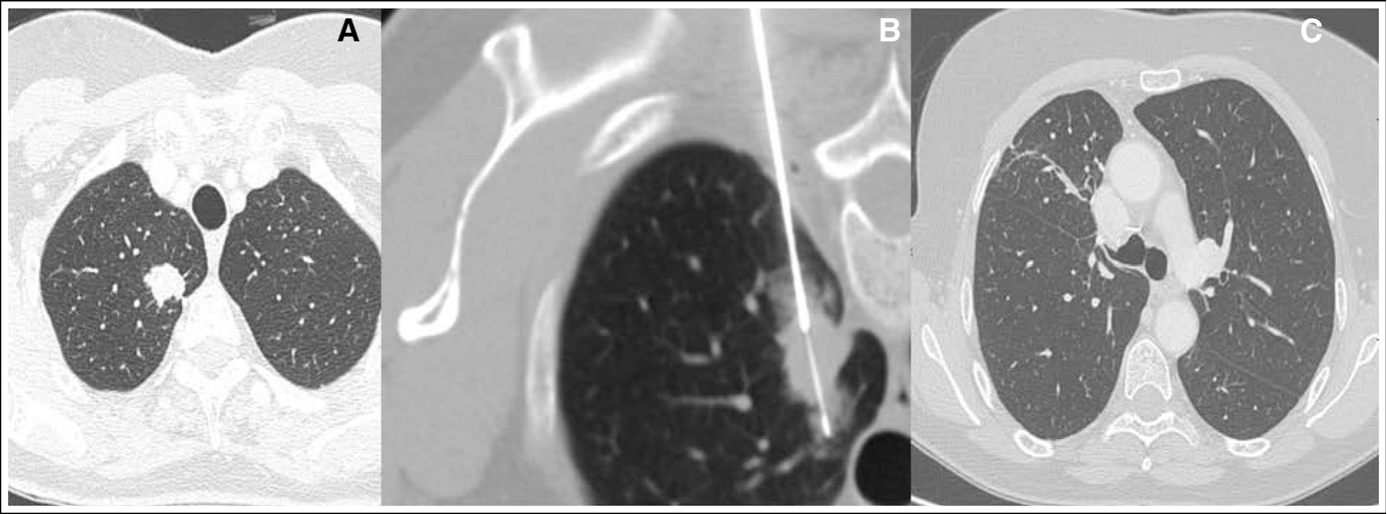
Computed tomography-guided pulmonary nodule biopsy performed on a patient included in the present study.

The majority of the PNs were found to be metastatic lesions from the primary tumor (64%).The remainder 36% were mostly either primary lung lesions (71.4% of nonmetastatic patients) or benign lesions (27.4% of nonmetastatic patients), as listed in Table 2. Figures 2 and 3 show two CT-guided PN biopsies performed in patients included in the present analysis. Of note, after the biopsy result, none of the study patients underwent rebiopsy. Among 24 patients with a primary breast cancer, we observed an immunohistochemistry profile difference between the primary breast tumor and the metastatic PN (primary tumor was negative for PR, and the metastasis was positive) in only one case. Patients with concomitant extrapulmonary nodules had a metastatic disease rate of 62.7%.

**Table 2:**
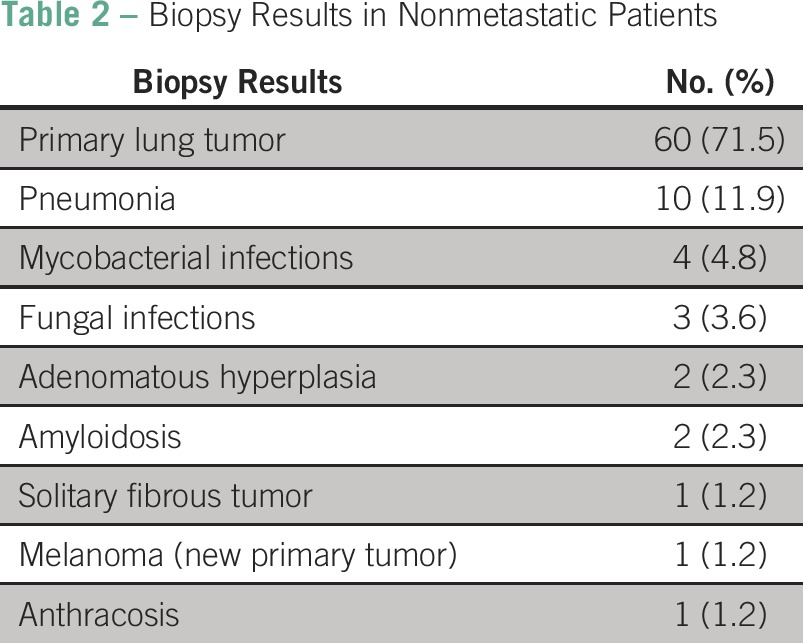
Biopsy Results in Nonmetastatic Patients

### Multivariate Analysis

Multivariable logistic regression analysis results are listed in Table 3. The presence of multiple PNs was significantly correlated with higher odds of finding metastatic disease on biopsy (OR, 5.08; 95% CI, 2.62 to 9.84; *P <* .01). Also, the presence of PN cavitation or signs of necrosis on CT scan was associated with statistically increased odds of finding metastatic disease (OR, 2.9; 95% CI, 1.03 to 8.21; *P =* .04). Of note, despite the small sample size, a PN in melanoma patients showed a trend toward increased odds of finding metastatic disease on biopsy, with an OR of 9.09 (95% CI, 0.83 to 99.73; *P =* .07). To evaluate which covariates were associated with a higher likelihood of finding metastatic disease on PN biopsy in patients presenting solely with PN, a similar analysis was performed, excluding patients who presented with concomitant extrapulmonary nodules. Interestingly, both multiple PNs and the presence of cavitation or necrosis were associated with metastatic disease to the lungs (OR, 4.24; 95% CI, 1.97 to 9.14; *P <* .01 and OR, 4.01; 95% CI, 1.24 to 13.01; *P* = .02, respectively). All other variables analyzed were not associated with higher odds of finding metastatic disease in the current study.

**Table 3:**
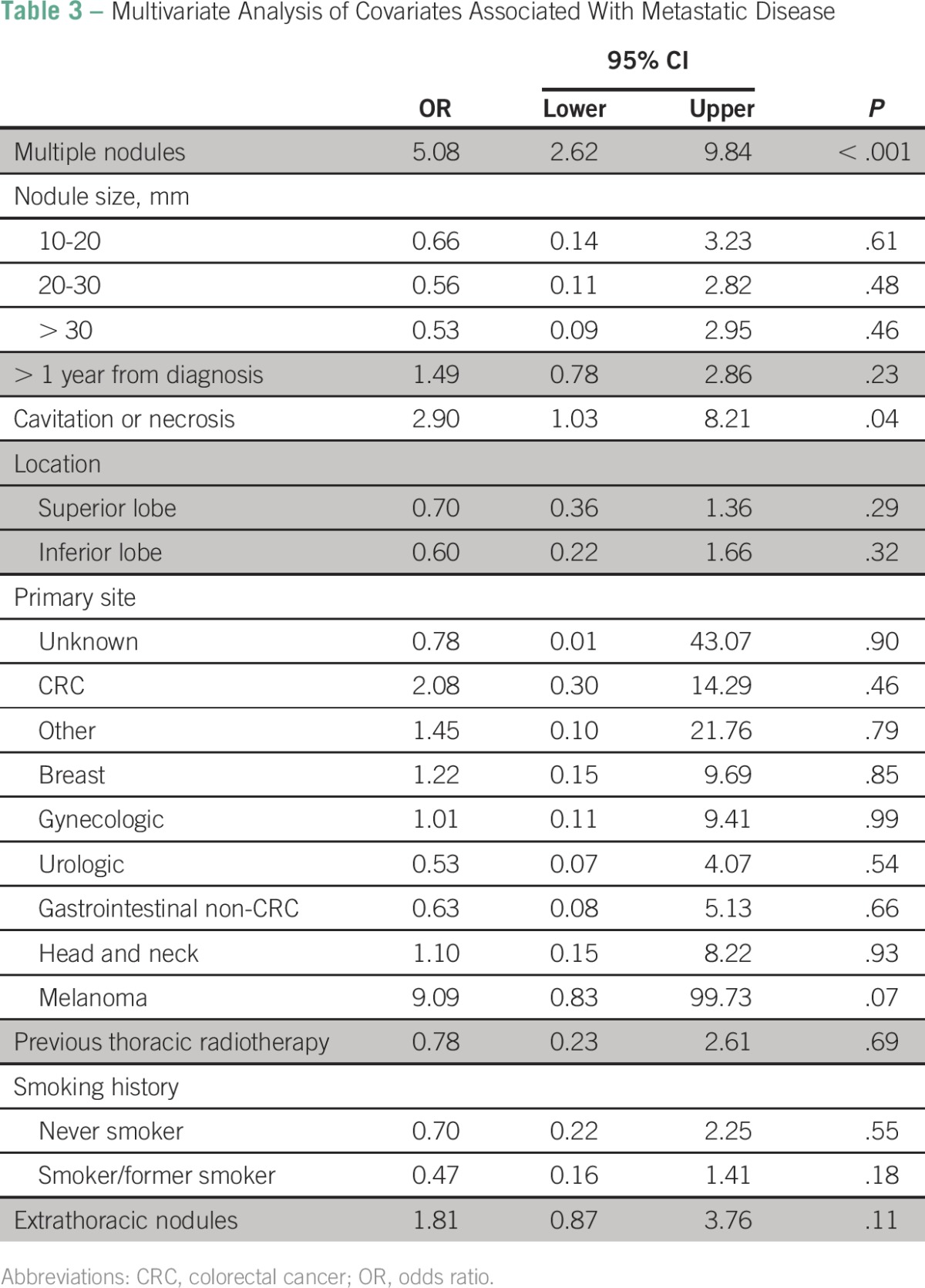
Multivariate Analysis of Covariates Associated With Metastatic Disease

### Procedure Complications

All patients underwent chest imaging (CT or x-ray) after biopsy. Of the 228 biopsies analyzed, 152 (66.6%) were uncomplicated. Among 76 complications, we observed one case of parenchymal hemorrhage and 75 cases of pneumothorax. Most pneumothoraxes (70) resolved spontaneously, and only five patients with pneumothorax required CT-guided percutaneous drainage for up to 5 days. The patient with parenchymal hemorrhage was monitored at the intensive care unit for 48 hours and was discharged. No patient required a blood transfusion, and there were no procedure-related deaths.

## DISCUSSION

In our sample of 228 patients with nonpulmonary cancer presenting with PNs, more than one third (36.9%) of the PNs were unrelated to patients’ primary tumor, including 9.6% of benign lesions, such as infectious diseases or anthracosis. The biopsy procedure has demonstrated to be safe, with only 2.6% of patients presenting clinically relevant complications after biopsy. Among the 36.9% of patients with nonmetastatic lesions, PN biopsy resulted in a radical change in the patient’s final diagnosis and therapeutic plan. Infectious diseases were treated with antimicrobial drugs, benign lesions were observed, and new primary cancers were staged and treated accordingly. If all PNs were presumed to be metastatic disease, more than one third of these patients would have received inappropriate treatment.

Although not within the scope of our study, we also observed that one of 24 patients with breast cancer presented a change in immunohistochemical profile: the primary tumor was estrogen receptor negative and PR negative, and the PN was PR positive. This finding has been previously reported and has clinical implications because it may provide an opportunity for the patient to receive more effective treatment for breast cancer recurrence.^9,10^

Our results are comparable to other series.^5-7^ In 1978, Cahan et al^5^ described 35-year thoracotomy results in more than 800 patients presenting with PNs and observed that the PNs represented primary lung cancer, metastases, or nonneoplastic lesions. Since then, efforts to better understand the etiology of PNs in patients with cancer have become necessary.

Other series have also found different rates of malignant nodules on histologic analysis of PNs of patients with cancer. In a study with 1,104 patients undergoing PN resection, Mery et al^6^ observed a 63% malignancy rate in 337 patients with previous cancer history. Their study included patients with lung cancer as well as patients without a cancer diagnosis, who were not included in our analysis. A lower rate of malignant PNs was found by Khokhar et al.^7^ In 151 patients with extrapulmonary cancers who underwent PN biopsy, 42% of the nodules were found to be malignant, including newly diagnosed lung cancers.

The presence of multiple PNs and cavitary/necrotic lesions were associated with higher odds of finding metastatic disease, regardless of the presence of concomitant extrapulmonary nodules. We did not find a significant association between the site of the primary tumor and higher odds of metastatic disease, as observed in a previous study.^11^ However, a trend toward increased odds of metastatic disease was shown for primary melanoma. The fact that this correlation did not reach statistical significance might be attributed to the relatively small number of melanoma patients (n = 15) included in our analysis.

Despite the associations observed, as a result of the high prevalence of nonmetastatic disease and the low incidence of procedure-related complications observed in the current study and in other series, PN biopsy should be routinely recommended for patients with nonpulmonary cancer presenting with PNs. Perhaps in patients expected to have a higher risk of lung biopsy complications, the presence of multiple and/or cavitated nodules could favor metastatic disease and guide therapy, although no established guidelines currently exist.

This analysis has some limitations because of its retrospective nature. Referral for PN biopsy was a choice of the patient’s clinician. Therefore, because this was an exploratory study, it was not possible to quantify the number of patients presenting with PNs who would be eligible for our study but who were not referred for biopsy, representing a possible selection bias. Additionally, this was a single-center analysis, in which the prevalence of infectious disease, such as tuberculosis, might be distinct from other centers worldwide. Although the procedural complication rate in this study was low and seemed acceptable and manageable, such occurrences are not trivial.^12^ Our institution is a high-volume tertiary-care institution with expertise in image-guided invasive procedures. The logistics involved in the procedure and related follow-up are complex. Our results only apply to institutions with such a profile.

Patients presenting with concomitant extrathoracic nodules detected on CT scans were included in the present analysis if they did not have biopsy-proven metastatic disease. This might represent a confounding factor because PNs in these patients are supposed to have higher odds of being metastatic disease. Interestingly, on multivariate analysis, the presence of concomitant extrathoracic lesions was not associated with higher odds of finding metastatic disease. Moreover, a subsequent multivariate analysis excluding this subgroup of patients demonstrated results similar to the first analysis. Of note, patients with non-neoplastic diseases, such as tuberculosis and fungal infections, can present with extrathoracic nodules. Given the small number of these patients in our study, it is difficult to draw definitive conclusions, but the results indicate that in patients presenting with PNs and concomitant extrathoracic nodules on CT scans, the nodules should not be assumed to be metastatic without biopsy confirmation. Further studies to address this question are necessary.

In conclusion, this study demonstrated that the majority of PNs observed in patients with cancer were metastases from the primary tumor. Despite this, more than one third of the patients did not have metastatic cancer. Percutaneous CT-guided PN biopsy is safe and can provide valuable information. Because no validated clinical tools exist to predict whether a PN is a metastasis, biopsy is recommended. Our data demonstrate that assuming that all PNs observed in patients with cancer are metastatic disease will lead to high rates of inaccurate diagnosis and inappropriate subsequent treatments. Tissue sampling is still fundamental for accurately diagnosing and treating patients with cancer.
